# Antioxidant and Antifungal Properties of Cinnamon, Cloves, *Melia azedarach* L. and *Ocimum gratissimum* L. Extracts against *Fusarium oxysporum* Isolated from Infected Vegetables in Mauritius

**DOI:** 10.3390/pathogens13060436

**Published:** 2024-05-22

**Authors:** Rajesh Jeewon, Shaan B. Pudaruth, Vishwakalyan Bhoyroo, Aadil Ahmad Aullybux, Kunhiraman C. Rajeshkumar, Abdulwahed Fahad Alrefaei

**Affiliations:** 1 Department of Health Sciences, Faculty of Medicine and Health Sciences, University of Mauritius, Réduit 80837, Mauritius; 2 Department of Agricultural and Food Science, Faculty of Agriculture, University of Mauritius, Réduit 80837, Mauritius; 3 National Fungal Culture Collection of India (NFCCI), Biodiversity and Palaeobiology (Fungi) Gr., MACS Agharkar Research Institute, G. G. Agarkar Road, Pune 411 004, Maharashtra, India; 4 Department of Zoology, College of Science, King Saud University, P.O. Box 2455, Riyadh 11451, Saudi Arabia; afrefaei@ksu.edu.sa

**Keywords:** *Fusarium oxysporum*, plant extracts, phytochemical, antioxidant, antifungal

## Abstract

Background: *Fusarium* species, a group of economically destructive phytopathogens, are poorly studied in Mauritius where agriculture holds much significance. Furthermore, the increasing popularity of organic farming has prompted interest in alternatives to chemical fungicides. Methods: After gaining an overview of *Fusarium* prevalence in Mauritius fields through a survey, the pathogen was isolated from infected crops and identified based on morphological and molecular characteristics. Methanol and water extracts were then prepared from *Melia azedarach*, *Ocimum gratissimum*, cinnamon and cloves before determining their phytochemical profiles. Additionally, the antioxidant and antifungal effects of different concentrations of aqueous extracts were assessed. Results: The isolate was confirmed as *Fusarium oxysporum*, and cloves inhibited its growth by up to 100%, especially at 60 and 90 g/L, with the results being significantly higher than those of the synthetic fungicide mancozeb. Over 50% inhibition was also noted for cinnamon and *Ocimum gratissimum*, and these effects could be linked to the flavonoids, phenols and terpenoids in the extracts. Conclusion: This study presented the aqueous extracts of cloves, cinnamon and *Ocimum gratissimum* as potential alternatives to chemical fungicides. It also confirmed the prevalence of *Fusarium* infection in Mauritius fields, thereby highlighting the need for additional studies on the pathogen.

## 1. Introduction

Fungi, a fascinating group of eukaryotic organisms that includes yeasts, molds and mushrooms, consists of an estimated 1.5–5 million species, of which only 120,000–150,000 have been described [1,2,3]. Being morphologically, metabolically and ecologically diverse, fungi are present in virtually all known habitats where they not only act as decomposers and food sources but also interact with other organisms through symbiotic, parasitic or pathogenic relationships [3,4,5]. Fungal pathogens, in particular, are of research interest as they are often responsible for diseases, including life-threatening ones, in humans. In addition, they also cause significant agricultural losses that can have long-lasting impacts, especially when staple crops are involved, with arguably one of the most popular examples being the Irish Great Famine triggered by *Phytophthora infestans*, the cause of potato late blight [6,7,8]. At the same time, as noted by different reports, lower crop yields and quality resulting from plant diseases represent a significant financial burden on farmers [6,9]. 

*Fusarium* species, of the Phylum *Ascomycota*, Subphylum *Pezizomycotina*, Class *Sordariomycetes*, Order *Hypocreales* and Family *Nectriaceae*, are arguably one of the most important plant fungal pathogens that occur worldwide [10,11]. Indeed, this group of over 70 recognised species have a very wide host range, infecting a number of important food and cash crops such as cotton, banana, maize and wheat, just to name a few, with the symptoms often including yellowing and wilting of leaves, root or stem rots, stunted growth, fruit and seed rots and vascular wilt [12]. As a result, *Fusarium* infection may cause significant yield loss, leading to social and economic consequences. In addition, many species can produce mycotoxins, and these secondary metabolites which accumulate in crop tissues can threaten human and animal heath when consumed in sufficient quantities [11,13]. Therefore, adequate control of *Fusarium* infection is a major requirement to minimise crop loss. 

However, in practice, management of *Fusarium* species is challenging as the fungi can survive a wide range of environmental conditions, can grow on various substrates and have a very efficient means of spore dispersal [12]. Furthermore, being soilborne phytopathogens, they can easily survive in soil and plant debris for several years, thereby facilitating their spread through the movement of infected soil by agricultural tools and/or animals [11,12]. Consequently, to mitigate the impact of diseases, integrated management strategies that combine different agricultural practices are often favoured, and these include the breeding of resistant crop varieties, the use of certified pathogen-free starting seeds, biological control using beneficial microorganisms, chemical control with fungicides, good agronomic practices (e.g., crop rotation) and early detection of infection, amongst others [14,15,16,17]. 

Mauritius is a tropical island that has a high incidence of fungal diseases. In this context, an early study by Dulymamode et al. [18] reported that over 200 taxa were identified on the island, of which 90% were new records that had been assigned to one genus and 38 new species. However, studies that specifically focus on *Fusarium* species are severely lacking, with the only work being a 2023 study by Mamode Ally et al. [19] who reported the first case of *Fusarium acacia-mearnsii* responsible for leaf blight in pumpkin in Mauritius. This highlights the need to undertake more research on the prevalence of *Fusarium* species in the country as an outbreak of *Fusarium* infection could have devastating consequences for Mauritius, which relies significantly on local agricultural produce to fulfil local needs for vegetables. As far as the control of fungal diseases is concerned, despite the efficacy of integrated practices, the widespread application of synthetic fungicides remains arguably the most popular approach for Mauritian farmers. However, it is widely recognised that the intensive use of chemical fungicides negatively impacts the environment, the soil, the plants and even human health [20,21]. In addition, the likelihood that fungi may develop resistance to the chemicals can, in fact, reduce the impact of such control measures. An increasing number of consumers are also now more conscious about the presence of chemical residues on crops, and altogether, these different factors have prompted a search for eco-friendly alternatives to synthetic fungicides. In this context, the potential of plant extracts as antifungal agents has been gaining popularity, with a survey of literature actually showing an increasing number of studies focused on this type of research. Plant extracts also consist of a mixture of different compounds and as such, they can exert antifungal activity through different mechanisms, thereby reducing the likelihood of having fungal resistance [22]. Furthermore, the government of Mauritius has been actively promoting organic and bio-farming schemes alongside regulations that seek to control and monitor the amount of pesticides used on fruits and vegetables. Therefore, in this case, the use of plant extracts as alternative means of fungal control would be in line the government’s objectives.

Based on the above, the aim of this study was to assess the extent of *Fusarium* infections in the vegetable crops of Mauritius before investigating the antioxidant and antifungal effects of cinnamon, cloves, *Melia azedarach* L. and *Ocimum gratissimum* L. extracts for the control of *Fusarium*. For this purpose, a survey was first performed among farmers to determine the prevalence of infection in their fields. Fungi were then isolated from infected vegetables and identified based on their morphological features as well as internal transcribed spacer sequences. Finally, the different plant extracts were prepared and assessed for antioxidant and antifungal properties along with phytochemical analysis of their constituents. It is expected that the results will be of significance to assess the suitability of the selected plant extracts as potential alternatives to chemical fungicides.

## 2. Materials and Methods

### 2.1. Survey on Damages Caused by Fusarium

A short survey was carried out in vegetable fields from different parts of Mauritius to assess the types of vegetables commonly affected by *Fusarium*, the most prevalent symptoms and the possible causative agents. Areas where the types of crops grown and the environmental conditions would likely be more conducive to the spread and growth of *Fusarium* species were selected for the survey.

### 2.2. Sample Collection

Infected potato samples were collected from a local vegetable market in Quartier Militaire (Moka district), while those of tomatoes were obtained from a field in Olivia (Flacq district). Both samples showed symptoms of black rot where the outer surface had turned “watery”, especially in the case of the tomatoes. In order to prepare extracts, four types of plant samples were also collected. These included *Melia azedarach* L., *Ocimum gratissimum* L. and cinnamon, obtained from different areas of Camp Thorel (Moka district) as well as cloves which were bought from a market in Melrose (Moka district).

### 2.3. Isolation of Fungi from Infected Samples

Fungi were isolated from the collected samples using the method of Ali et al. [23], with some modifications. Briefly, the infected vegetables were dipped in 10% sodium hydrochlorite solution for 30–60 s, after which they were rinsed three times with sterilised distilled water. The samples were then placed in a laminar flow where they were cut into pieces of size 3 × 3 mm using sterile scalpels and forceps before being transferred to petri dishes containing potato dextrose agar (PDA, Himedia, Mumbai, India) that had been supplemented with chloramphenicol (0.25% *w*/*v*, Himedia, Mumbai, India). After sealing the plates with parafilm, incubation was performed at room temperature for seven days in the dark, with the plates regularly observed under a light microscope to monitor fungal growth. For each vegetable sample, there were five replicates. 

### 2.4. Single Spore Isolation

Single spores were obtained by dilution plating. Using a loop, a small part of each isolated fungus was suspended in 10 mL of sterilised water in a test tube. A loopful of the resulting suspension was subsequently streaked onto PDA agar and left overnight at room temperature. Conidial germination was observed under a microscope and after being removed with a sterile scalpel, the cut part was placed on fresh PDA media to obtain pure fungal cultures. The plates were regularly viewed under a microscope using cotton blue lactophenol. Pure cultures were maintained through weekly subculturing which involved cutting a 5-mm piece across the edge of the plates with a sterile scalpel and its subsequent transfer to a new agar plate.

### 2.5. DNA Extraction

DNA extraction from the isolated fungi was based on the method of Umesha et al. [24], with modifications. Briefly, 0.5 g of mycelium was ground in liquid nitrogen before adding 5 mL of hot (60 °C) 2× cetyltrimethyl ammonium bromide (CTAB, Sigma-Aldrich, St. Louis, MI, USA) buffer, 0.2% mercaptoethanol (Fisher Scientific, Hampton, VA, USA) and 2% PVP (Fisher Scientific, Hampton, VA, USA). This was followed by incubation in a 60 °C water bath for 25–30 min with occasional swirling, after which two-thirds volume of chloroform-isoamyl alcohol (24:1) was added. The mixture was centrifuged for 15 min at 2000 rpm and after removing the upper aqueous layer, the chloroform-isoamyl alcohol extraction was repeated until no interface was observed. NaCl (0.1 volume; 5 M) was then added along with ice-cold isopropanol (2/3 volume), with the resulting solution incubated at −20 °C. The precipitated DNA was collected by centrifugation for 30 min at 200 rpm, and after washing the pellet with 70% ice cold alcohol, it was dissolved in 0.5 mL of sterile water.

### 2.6. PCR, Sequencing and Analysis

The quality of the extracted DNA was assessed spectrophotometrically based on the ratio of absorbances recorded at 260 and 280 nm. For this purpose, the DNA (10 µL) was first dissolved in 990 µL of sterile water before recording the absorbance values using sterile water as blank. The DNA was subsequently amplified by PCR (ITS 4 and ITS 5) before being sent to Inqaba Biotechnical Laboratories (Pty) Ltd. (Pretoria, South Africa) for sequencing. The resulting sequences were cleaned in BioEdit (version 7.2.5), and once the consensus sequence was obtained, it was queried against the NCBI database using BLAST. The sequence was submitted to NCBI under the accession numbers PP494541 and PP494542.

Validly published organisms showing closest matches as well as those from other *Fusarium* species were then selected and aligned to the query sequence using ClustalW [25], with phylogenetic trees subsequently constructed in PAUP 4.0 [26] using the neighbour-joining—NJ [27]—and maximum likelihood—ML [28]—methods to determine the relationship of isolated species with other organisms. For NJ, distance matrices were first generated according to Kimura’s 2-parameter model [29], while for ML, the best evolutionary model was selected as determined by jModelTest [30,31]. The robustness of tree topologies was subsequently evaluated using bootstrap analysis with 1000 and 100 replications for NJ and ML, respectively. A phylogenetic tree showing nodes which were common between the two methods was eventually presented to highlight strong support for relationships between taxa.

### 2.7. Preparation of Crude Extracts

Fresh leaf samples were brought to the lab where they were washed thoroughly under running tap water, and after being air dried for 1 h, they were ground separately in an electric grinder. To 100 g of each powdered leaf sample in a conical flask, 300 mL of methanol or water was added. A cotton plug and aluminium foil were then placed at the top of each flask to prevent evaporation, after which the flasks were kept on a shaker at room temperature to allow the extraction process to take place. After seven days, the extracts were filtered and concentrated in a rotary evaporator at 60 °C and 75 rpm. The resulting methanol extracts were dissolved in dimethyl sulphoxide (DMSO, Himedia, Mumbai, India), while in the case of the water extracts, they were dissolved in water itself prior to storage at 4 °C. Based on the results, the extraction yield (% Yield) for the extracts was calculated as follows:

Yield (%) = (W1 × 100)/W2(1)

where W1 is the final weight of the extract, and W2 is the initial weight of the plant powder.

### 2.8. Phytochemical Analysis

Qualitative phytochemical analysis was performed, according to established methods [32,33,34,35], to determine the composition, especially the secondary metabolites content, of the different extracts. In this case, the presence of alkaloids, flavonoids, saponins, tannins, phenols, proteins, carbohydrates, cardiac glycosides and terpenoids were tested according to established methods. The procedures followed for each test are provided as Supplementary Information (Table S1).

### 2.9. Antioxidant Capacity Assays

#### 2.9.1. Ferric Reducing Antioxidant Power (FRAP) Assay

The methanol and water extracts (2 μL dissolved in 998 μL of DMSO, with the final volume subsequently made up to 10 mL with DMSO) were mixed with phosphate buffer (0.2 M, pH 6.6, 1 mL) and potassium hexacyanoferrate [K_3_Fe(CN)_6_] (1%, 1 mL) prior to a 20 min incubation at 50 °C. This was followed by the addition of trichloroacetic acid solution (10%, 1 mL) and after centrifugation for 15 min at 2000 rpm, the resulting supernatant was mixed with distilled water (1.5 mL) and ferric chloride (FeCl_3_) solution (0.1%, 0.1 mL). A second incubation was then performed for 10 min before measuring absorbance values at 700 nm. DMSO was used as a control for this experiment.

#### 2.9.2. DPPH Radical Scavenging Activity

The free radical scavenging activities of the different extracts were determined based on the 2,2-diphenyl-1-picrylhydrazyl (DPPH, Sigma-Aldrich, St. Louis, MI, USA) assay as described by Shirazi et al. [36], with modifications. DPPH solution was freshly prepared by dissolving 0.004 g of DPPH in 160 mL of methanol, and its absorbance was recorded at 515 nm. For the extracts, 50 μL was mixed with 3 mL of DPPH solution and after vortex mixing, the resulting solution was incubated at room temperature for 15 min in the dark. Absorbance values were eventually measured at 515 nm and recorded. 

#### 2.9.3. Antifungal Assay

Three of the four plant samples, namely *Melia azedarach* L., *Ocimum gratissimum* L. and cinnamon were washed thoroughly under tap water and allowed to dry for 1 h in open air, while clove samples were placed in an oven at 60 °C. The plants were then separately grounded into powdered form and for each sample, different amounts (30, 60 and 90 g) were added to 1000 mL of sterilised distilled water to prepare mixtures of different concentrations. These mixtures were kept in a water bath at 100 °C for 1 h and subsequently allowed to stand overnight before being filtered through one layer of muslin cloth to yield the final extracts. Forzeb 80 WP (Mancozeb, UPL Limited, Mumbai, India), a chemical fungicide was also used as positive control, and in this case, different concentrations were prepared using 4, 8 and 12 g/L.

For the antifungal assay, 25 mL of each extract was added to 75 mL of sterilised PDA in a conical flask, and the resulting mixture was poured into five petri plates. A negative control, whereby the extract was replaced by 25 mL of sterilised distilled water, was also included. A cork-borer was then used to punch holes (10 mm in diameter) across the edge of plates containing 10-day-old fungi, with one of the cut pieces subsequently placed in the centre of each plate containing the media–extract mixture. Fungal growth was observed for seven days to determine the efficacy of the different extracts against *Fusarium oxysporum*, and any visible growth was measured with a ruler to calculate the percentage of growth inhibition (PGI) as follows:

(2) $$PGI\;(\%)=\frac{R-R1}{R}\times 100$$

where *R* is the distance (measured in mm) from the inoculation point to the colony margin in control plates; *R*1 is the distance of fungal growth from the point of inoculation to the colony margin in treatment plates. All results were statistically analysed using Analysis of Variance, followed by Tukey’s post hoc tests at 5% significance level (*p*-value < 0.005). Pairwise comparisons were also performed between groups to identify significantly different results.

## 3. Results

### 3.1. Prevalence of Disease

Most of the survey was conducted in La Laura region (Moka district) in the centre of Mauritius during the summer and winter seasons. Out of 25 farmers, 5 stated that less than 10% of their crops were affected, while 10 mentioned that 20–50% of their crops were affected every year due to fusarium wilts. Furthermore, for six farmers, 50–75% of the crops were damaged the previous year, with the four remaining farmers confirming an outbreak of fusarium wilt that resulted in the loss of more than 80% of their crops. The farmers used different chemical fungicides to control the disease, but this approach was not considered as being effective. Overall, tomatoes were found to be the most affected, with the main symptoms being fusarium wilt, black rot, yellowing of leaves and stunted growth. Furthermore, the disease was more prevalent in summer, with the main causative agent likely being *Fusarium oxysporum* f.sp. *lycopersici*. Detailed results of the survey are available as Supplementary Information (Table S2).

### 3.2. Isolation and Morphological Characteristics of Fungi

**Figure 1:**
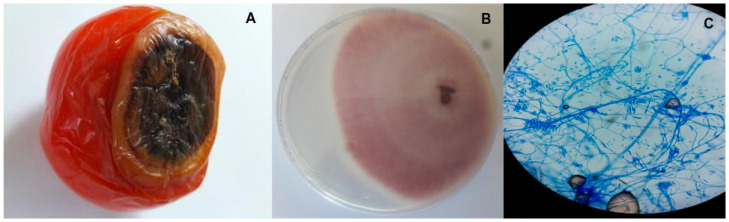
(**A**) Rotting of tomato tissues due to fusarium wilt. (**B**) Pure culture of *Fusarium oxysporum* on PDA. (**C**) Microscopic view of microconidia, macroconidia and chlamydospores of *Fusarium oxysporum*.

According to Sanchez et al., [37], *F*. *oxysporum* f. sp. *lycopersici* causes fusarium wilt in tomatoes; hence, infected vegetables were selected based on the symptoms described (Figure 1A). The same procedure was applied when selecting the potato samples. Pure fungal cultures (Figure 1B) obtained from the infected vegetables were regularly observed under a microscope using cotton blue lactophenol (Figure 1C) and the colonies were identified as *Fusarium oxysporum* based on morphological features such as mycelial colour, mycelial growth, sporulation, formation of chlamydospores and conidial size [38].

Specifically, the isolated fungal colonies showed significant variations in colour, with most of the plates having violet to peach colonies, which fitted the description of Booth [39]. The microconidia had one or no septate, were cylindrical or oval in shape and were seen on short monophialides, while in terms of size, they varied between 2.6 and 12 μm × 2.0 and 3.8 μm (length × breadth) among the different isolates. In addition, short to medium and usually straight macroconidia with three to five septate were observed, with the size being 19–39.5 μm × 2.2–4 μm (length × breadth). In the intercalary chains, the presence of chlamydospores was also noted in 4–5-day-old plates. Finally, the isolates exhibited quick growth at the incubation temperature, with the colony diameters reaching 70–75 mm in seven-day-old plates. Altogether, these morphological features fit the characteristics of *Fusarium* described by Nelson et al. [40].

### 3.3. Phylogenetic Analysis of the Isolates

**Figure 2:**
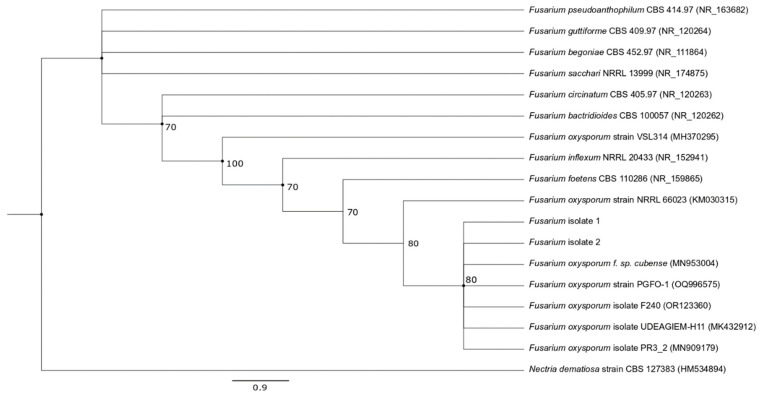
Phylogenetic tree of ITS sequences, based on NJ and ML trees, showing the relationship of the isolates with other *Fusarium* species. Bootstrap values (expressed as percentages of 1000 replications) ≥ 70 are shown at branch nodes and circles indicate nodes that were common to both NJ and ML trees. *Nectria dematiosa* strain CBS 127383 was chosen as the outgroup. Bar represents 0.9 substitution per nucleotide position.

Based on the morphological features and the sequencing results, the isolates were identified as *Fusarium oxysporum*, with their sequences showing 100% similarity to those of other isolates. Phylogenetic analysis further confirmed these findings as the isolated fungi formed a distinct cluster with *F*. *oxysporum* obtained from different sources, while being clearly separated from other *Fusarium* species with relatively strong bootstrap support (Figure 2).

### 3.4. Preparation of Plant Extracts

The extraction yield was compared for the four plants as well as for the two solvents (i.e., water and methanol). Overall, the crude methanol extract had a higher yield than that of water, probably due to the former’s higher polarity. Similarly, of all plants, cloves yielded the most extract, followed by cinnamon, *O*. *gratissimum* and *M*. *azedarach* in decreasing order of yield. A summary of the results is provided as Supplementary Information (Figure S1).

### 3.5. Phytochemical Analysis

The phytochemical constituents of the different plant extracts were determined. Overall, the methanol extracts of cinnamon and cloves contained all the tested phytochemicals and a nearly similar result was obtained for their water extracts, with the only exception being the absence of cardiac glycosides in the case of cloves. In contrast, a number of phytochemicals were not detected in the extracts from *M*. *azedarach* and *O*. *gratissimum*. For instance, they did not contain any flavonoids or saponins, while alkaloids were absent when water was used as the extraction solvent. In addition, phenols were only present in the methanol extract of *M*. *azedarach*, while cardiac glycosides were absent only from its water extract. These results are summarised in Table 1.

### 3.6. Antioxidant Activity of Extracts

The FRAP assay was used to determine the total antioxidant activity of the samples, and in this case, phenolic and flavonoid acids form coloured complexes with metal atoms such as iron and copper due to their strong antioxidant activity. As shown in Table 2, absorbance values measured at 700 nm suggested that the water extract of *M*. *azedarach* displayed the highest antioxidant activity, followed by the water extract of cloves. Similarly, the DPPH assay was used to determine the extracts’ ability to scavenge free radicals, with lighter colours of the DPPH solution indicative of stronger antioxidant activities. The results showed that the water extract of *M*. *azedarach* had the highest DPPH scavenging activity, followed by that of *O*. *gratissimum*, while, in contrast to the FRAP assay, cloves’ water extract had the lowest free radical scavenging activity (Table 2).

### 3.7. Antifungal Assay

For this assay, there was considerable variation in the growth of *F*. *oxysporum* on the agar-extract media. In most plates, there was uniform fungal growth, with the colour of the isolate varying between yellow, white, dark purple and light purple. However, in some cases, irregular growth patterns were also observed. Furthermore, the fungi had overgrown on some of the plates within seven days, while in others, overgrowth took longer than the seven-day incubation period. Interestingly, the absence of significant fungal growth was also observed in some plates, and these results, presented in Table 3, reflected the inhibitory effects of some of the extracts. 

**Figure 3:**
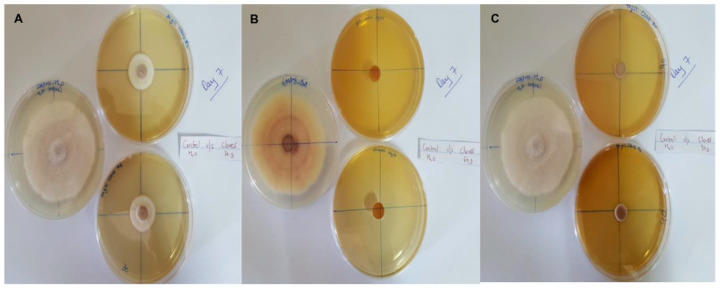
Antifungal activity of (**A**) 30 g/L, (**B**) 60 g/L and (**C**) 90 g/L of cloves extract compared with the negative control.

**Figure 4:**
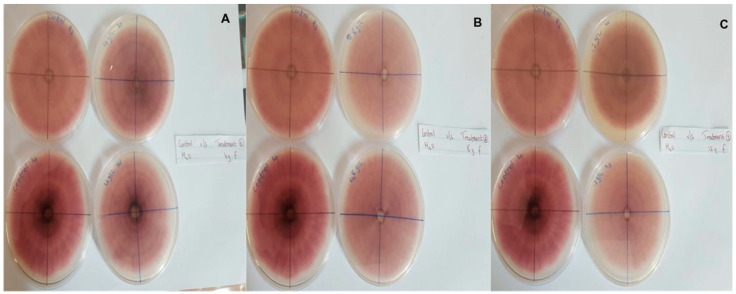
Antifungal activity of (**A**) 4 g/L, (**B**) 8 g/L and (**C**) 12 g/L of mancozeb compared with the negative control.

**Figure 5:**
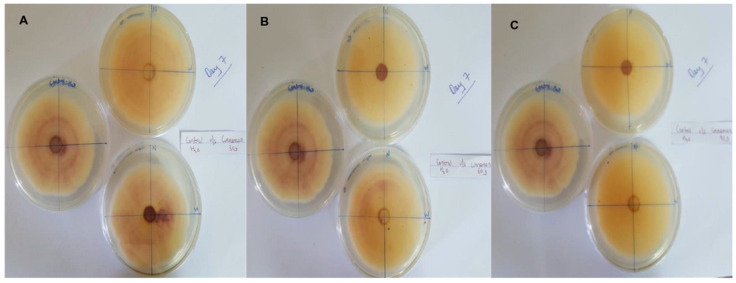
Antifungal activity of (**A**) 30 g/L, (**B**) 60 g/L and (**C**) 90 g/L of cinnamon extract compared with the negative control.

**Figure 6:**
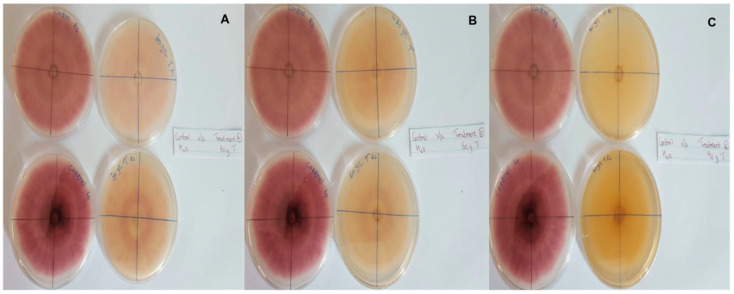
Antifungal activity of (**A**) 30 g/L, (**B**) 60 g/L and (**C**) 90 g/L of O. gratissimum extract compared with the negative control.

**Figure 7:**
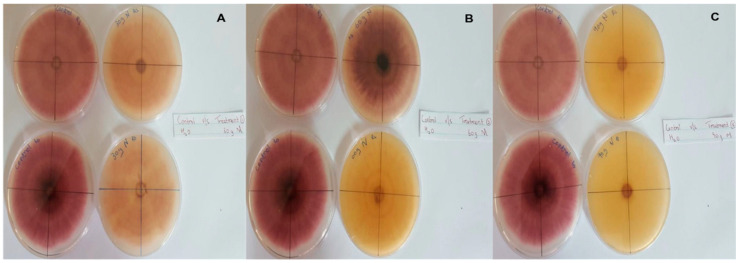
Antifungal activity of (**A**) 30 g/L, (**B**) 60 g/L and (**C**) 90 g/L of M. azedarach extract compared with the negative control.

In this context, clove extracts were found to exert the greatest antifungal activities (*p* < 0.05), with 100% of growth inhibition at all concentrations after 24 h of incubation (Figure 3). This value decreased to 78.6% after 168 h of incubation at an extract concentration of 30 g/L, although total inhibition was still observed when using higher concentrations irrespective of the incubation period. In fact, it is worth noting that these inhibitory effects were even significantly higher than those obtained from the chemical fungicide mancozeb (*p* < 0.05; Figure 4). Antifungal effects were also noted in the case of cinnamon (Figure 5) and *O*. *gratissimum* (Figure 6) extracts, for which the PGI varied between 29.9 and 59.7% at 24 h before decreasing to 7.1–15.3% at 168 h. In addition, the results revealed the low potency of *M*. *azedarach* extracts as antifungal agents (Figure 7). Furthermore, comparing the effects of extract concentration on the PGI across the different plants indicated that, even though greater inhibition was generally noted at higher concentrations, this difference was not significant (*p* > 0.05). In contrast, longer incubation times led to significantly lower antifungal activities (*p* < 0.05).

## 4. Discussion

### 4.1. Phylogenetic Analysis of Fusarium Isolates

In this study, infected vegetables were collected and the causal pathogen was isolated and identified before assessing the suitability of various plant extracts as potential antifungal agents. The vegetables were initially selected based on symptoms which were indicative of *Fusarium* infection [20,41] and, as shown in the results, the identity of the isolates was confirmed as *Fusarium oxysporum*. However, it is worth noting that the isolated fungi formed a distinct clade with *F*. *oxysporum* strains obtained from different hosts, including banana, guava and soybeans, amongst others. The ability of this pathogen to infect a wide range of plants is well documented in the existing literature, but individual strains are also known to be host-specific (*formae speciales*), especially due to distinct morphological and genetic features [42,43,44]. In this case, the absence of clear demarcations between the different strains could be attributed to the molecular marker used for identification. Indeed, *F*. *oxysporum* strains have a highly complex phylogeny and as such, are often referred to as being part of a *Fusarium oxysporum* species complex (FOSC) which group together closely related strains. Although sequencing of the ITS region is considered a suitable choice for distinguishing between members of this complex [45,46], O’Donnell et al. [47] provided a contrasting view, highlighting the limitation of the ITS sequences for this purpose. 

However, despite the potential influence of the selected marker on the results, the likelihood that the two isolated *F*. *oxysporum* strains could actually have a broad host range cannot be excluded. In this context, it has been noted that the horizontal transfer of chromosomes as well as homologous recombination can occur between members of the FOSC [48,49], and according to Ma et al. [50], such transfers can involve genes associated with host specificity, thereby leading to the emergence of pathogens with a wider host range. A broad host range can, in fact, be of particular concern for a country such as Mauritius where agriculture is not only of economic significance but also serves to fulfil local demands for vegetables and fruits. Future research should, therefore, consider the isolation and characterisation of pathogens from other food crops in view of assessing their relationship with the current isolates, with the use of additional established markers such as the translation elongation factor 1-α (*TEF1*), the β-tubulin and the DNA-directed RNA polymerase II largest (*RPB1*) and second largest subunit (*RPB2*) for molecular-based identification [46,47] also warranted.

### 4.2. Extent of Fusarium Infection in Vegetable Fields

The survey undertaken as part of this study to assess the prevalence of *Fusarium* infection indicated no specific patterns for the summer and winter months, with crops being still susceptible to the pathogen irrespective of the season. As far as Mauritius is concerned, being a tropical country, differences in temperature between summer and winter are less significant in comparison with non-tropical ones [51,52,53]. Similarly, even though winter months tend to be drier, rainfall is still relatively common, resulting in humid conditions. Hence, given that the severity of *Fusarium* infections is more severe in warm and humid weather [54,55], this would explain the disease prevalence reported by the farmers across the two seasons. 

A major limitation of this survey is that it was largely limited to a single region, namely La Laura in the centre of the country. However, since the purpose was to gain initial insight into the presence of *Fusarium* in fields and not to accurately map its prevalence, the results were deemed to be of significance to highlight the presence of this pathogen in Mauritius fields and, by extension, suggest potential infection by the same species in other parts of the island. Altogether, these findings highlight the need to establish appropriate control measures throughout the year to minimise crop loss, and such measures may even take on greater importance in the future as a result of changing climatic conditions which are likely to favour warmer climate, thereby promoting the prevalence of *Fusarium* infection [56,57].

### 4.3. Biological Activities of Plant Extracts

Two main biological activities, namely antioxidant and antifungal, were investigated for the different plant extracts used in this study. The results of the FRAP and DPPH radical scavenging assays revealed variations in the antioxidant activities of the extracts and this could be attributed to differences in their phytochemical constituents. This potential can, in fact, be of significance to crops as the exogenous application of antioxidants has been reported to improve plant health, resilience to stress or even influence endogenous antioxidant systems which are linked to defence mechanisms [58]. For instance, under dry conditions, the foliar application of clove fruit extract and/or salicylic acid to potato tubers was shown to enhance growth characteristics as well as defensive antioxidant components, with similarly enhanced features noted when folic acid, ascorbic acid and salicylic acid were sprayed on the foliage of potato under salt stress conditions [59,60]. Different reports have highlighted a link between plant stress and pathogen infection [61,62], and, therefore, even though the antioxidant plant extracts in this study may not directly confer protection against plant pathogens, enhanced stress tolerance in response to their application may subsequently translate into lower susceptibility to the pathogens.

In terms of antifungal properties, the extracts from *Melia azedarach* L. seemed to be particularly ineffective in controlling *Fusarium* growth even at the highest concentration of 90 g/L. In contrast, the clove extracts achieved total inhibition of fungal growth over the seven-day incubation period, especially at 60 and 90 g/L. The ability of these extracts to inhibit fungal growth is already well established, but a number of research studies have actually been focused on essential oils extracted using organic solvents, with the phenolic compound eugenol considered to be responsible for the activity [63,64,65]. However, in this study, a water-based extraction method was used and as reported in the review of Gengatharan and Rahim [66], eugenol was less likely to be present, being mostly detected in non-aqueous extracts. Hence, even though a detailed analysis of the extracts’ constituents was not undertaken, it seemed plausible that other compounds could have contributed to the observed antifungal effects. In this context, Li et al. [67] identified glucuronides, glycosides and acid compounds such as chlorogenic acid when studying aqueous extracts of cloves, while Hassan et al. [68] found myricetin, quercetin, kaempferol and ellagic acid to be present, amongst other compounds. Interestingly, many of these compounds are known to exhibit antifungal properties [69,70,71], with Hassan et al. [68] even reporting the application of clove water extract to reduce the severity of *Alternaria* fruit rot after harvest. These findings were consistent with the results of phytochemical analysis, which confirmed the presence of flavonoids and phenols in the extracts. Nevertheless, in-depth characterisation of the clove extract is warranted as this would be useful to identify the compounds which inhibit fungal growth the most, in view of their potential application as fungicides.

It is worth noting that the antifungal effects of the clove extracts were significantly better (*p* < 0.05) than those obtained at the maximum concentration of Mancozeb, thus highlighting cloves’ potential as an alternative to existing chemical fungicides. In addition, water-based extracts provide a number of benefits in terms of their non-toxicity, non-persistence in soil, biodegradability and ease of use, especially since, unlike extracts obtained from organic solvents, an appropriate formulation is not required prior to application to plants [72].

The next most effective extract for controlling fungal growth was that of cinnamon, followed by that of *Ocimum gratissimum* L. The suitability of aqueous cinnamon extracts as antifungal agents has been reported before [73,74] and, in fact, was not surprising given that they had a nearly similar phytochemical profile as that of clove extracts. However, despite the presence of similar classes of compounds, differences in terms of actual composition could be responsible for the lower activity against fungi, although this is yet to be assessed. As far as the extracts from *O*. *gratissimum* L. are concerned, in contrast to what has been reported before [75], the current results showed that they lacked saponins, alkaloids and flavonoids, which are known to inhibit fungal activity. Yet, surprisingly, their antifungal effects were not significantly different from those of cinnamon (*p* > 0.05). In this case, the inhibitory effects on fungal growth could potentially be attributed to terpenoids, such as monoterpenes and sesquiterpenes, which, according to the report of Mohr et al. [76], can exert their activity through hydrogen bonds with the active sites of target enzymes. Despite the above promising results, it is worth pointing out that this work was largely focused on water-based extracts. Given that phytochemical analysis revealed the presence of more constituents in methanol-based extracts, it would be useful to also assess their antifungal potential, especially in comparison with the current findings.

## 5. Conclusions

This work provided an overview of the prevalence of *Fusarium* infection in Mauritius fields before highlighting the antioxidant and antifungal properties of different plant extracts. In particular, the results established the water-based extracts of cloves, cinnamon and *Ocimum gratissimum* L. as potential alternatives for controlling fungal diseases. However, this study was not without limitations. Firstly, the survey was limited in scope and a more extensive one that covers more regions could help to accurately picture disease prevalence across the country. Furthermore, despite the isolate being identified as *Fusarium oxysporum*, the phylogenetic relationship could not be resolved and, in this case, the use of additional markers for subsequent studies is encouraged to achieve greater accuracy in identification, especially at the sub-species level, as such information would help to establish the susceptible host range of the pathogen. The potential of the methanol-based extracts as antifungal agents also needs to be assessed as their phytochemical profiles suggest that they might also exhibit promising biological activities. Finally, while the results do provide an overview of the phytochemicals which could be linked to the observed activities, an in-depth characterisation of the extracts would still be required to identify and possibly isolate the active compounds. Overall, this work indicates that *Fusarium oxysporum* species are present in Mauritius fields and with research on the pathogen still lacking, it serves as a reminder of the need to monitor its prevalence to mitigate any risk of severe losses to the country’s agricultural sector.

## Figures and Tables

**Table 1:** Phytochemical analysis of extracts from *Melia azedarach* L., *Ocimum gratissimum* L., cinnamon and cloves.

Plant Chemical Constituent	Methanol-Based Extraction				Water-Based Extraction			
	*Melia azedarach* L.	*Ocimum gratissimum* L.	Cinnamon	Cloves	*Melia azedarach* L.	*Ocimum gratissimum* L.	Cinnamon	Cloves
Flavonoids	−	−	+	+	−	−	+	+
Saponins	−	−	+	+	−	−	+	+
Tanins	+	+	+	+	+	+	+	+
Phenols	+	−	+	+	−	−	+	+
Proteins	+	+	+	+	+	+	+	+
Alkaloids	+	+	+	+	−	−	+	+
Cardiac Glycosides	+	+	+	+	−	+	+	−
Terpenoids	+	+	+	+	+	+	+	+
Carbohydrates	+	+	+	+	+	+	+	+

Key: − = absent + = present.

**Table 2:** Antioxidant activities of the different extracts as determined by the FRAP and DPPH assays.

		Ferric Reducing Antioxidant Power Assay (FRAP)	DPPH Radical Scavenging Activity
Methanol-based extraction	*Melia azedarach* L.	0.119	0.338
	*Ocimum gratissimum* L.	0.159	0.209
	Cinnamon	0.260	0.050
	Cloves	−0.103	0.081
Water-based extraction	*Melia azedarach* L.	0.434	0.601
	*Ocimum gratissimum* L.	0.292	0.697
	Cinnamon	0.107	0.396
	Cloves	0.333	0.008

**Table 3:** Effects of different concentrations of plant filtrates on the percentage growth inhibition of *Fusarium oxysporum* over a seven-day incubation period. The chemical fungicide Mancozeb acted as the positive control. The superscript shows results that were similar to that of the negative control.

Plant Extract	Amount Used (g) Per L	Incubation Period (h)						
		24	48	72	96	120	144	168
*Melia azedarach* L.	30	16.4 ± 4.8	4.9 ± 0.4 ^a^	6.9 ± 6.5 ^a^	5.3 ± 5.0 ^a^	3.6 ± 3.0 ^a^	2.1 ± 0.8 ^a^	1.4 ± 0.02 ^a^
	60	16.4 ± 4.7	4.9 ± 0.4 ^a^	6.9 ± 6.5 ^a^	5.3 ± 5.0 ^a^	3.6 ± 3.0 ^a^	2.2 ± 0.8 ^a^	1.4 ± 0.01 ^a^
	90	16.4 ± 4.7	4.9 ± 0.4 ^a^	4.9 ± 3.1 ^a^	2.4 ± 0.1 ^a^	1.8 ± 0.1 ^a^	1.6 ± 0.03 ^a^	1.4 ± 0.02 ^a^
*Ocimum gratissimum* L.	30	29.9 ± 8.7	19.4 ± 6.9	18.9 ± 8.5	15.3 ± 7.9	13.4 ± 4.3	10.8 ± 3.9	7.1 ± 1.4 ^a^
	60	39.2 ± 14.9	27.7 ± 3.6	28.3 ± 0.9	21.0 ± 3.1	19.0 ± 3.6	21.8 ± 1.8	12.4 ± 0.9
	90	55.9 ± 10.1	30.7 ± 8.8	37.3 ± 2.4	27.3 ± 2.0	21.5 ± 0.7	23.3 ± 1.8	15.3 ± 1.9
Cloves	30	100.0 ± 0.00	91.7 ± 3.3	87.9 ± 0.8	87.4 ± 2.1	83.4 ± 0.5	81.4 ± 0.4	78.6 ± 0.3
	60	100.0 ± 0.0	100.0 ± 0.0	100.0 ± 0.0	100.0 ± 0.0	100.0 ± 0.0	100.0 ± 0.0	100.0 ± 0.0
	90	100.0 ± 0.0	100.0 ± 0.0	100.0 ± 0.0	100.0 ± 0.0	100.0 ± 0.0	100.0 ± 0.0	100.0 ± 0.0
Cinnamon	30	56.7 ± 3.0	32.8 ± 1.9	27.2 ± 1.4	28.0 ± 2.7	18.3 ± 3.4	18.7 ± 0.4	8.7 ± 6.5
	60	56.7 ± 2.9	31.2 ± 5.9	23.2 ± 2.3	26.6 ± 2.9	18.4 ± 3.3	13.9 ± 3.8	10.4 ± 2.9
	90	59.7 ± 4.0	31.4 ± 8.7	28.2 ± 2.3	31.9 ± 3.9	14.9 ± 11.4	13.6 ± 10.7	8.6 ± 6.6
Mancozeb (control)	4	16.1 ± 9.7	14.5 ± 8.0	12.8 ± 8.3	12.4 ± 4.3	10.3 ± 5.9 ^a^	9.2 ± 5.4 ^a^	4.7 ± 2.9 ^a^
	8	20.1 ± 2.0	8.0 ± 4.9 ^a^	10.8 ± 8.5 ^a^	9.1 ± 7.9 ^a^	9.7 ± 7.3 ^a^	12.3 ± 6.4	8.6 ± 5.1
	12	60.4 ± 6.1	14.3 ± 8.7	19.4 ± 5.8	16.8 ± 8.4	16.57 ± 0.4	15.5 ± 4.0	10.5 ± 2.9

## Data Availability

No new data were created or analysed in this study. Data sharing is not applicable to this article.

## Supplementary Materials

The following supporting information can be downloaded at: https://www.mdpi.com/article/10.3390/pathogens13060436/s1, Figure S1: Extraction yield of different plants using methanol or water as solvent; Table S1: Tests for the different constituents present in the extracts of cinnamon, cloves, *Melia azedarach* L. and *Ocimum gratissimum* L.; Table S2: Results of the survey to assess the prevalence of *Fusarium* infection in Mauritius fields.
