# Supplementary material for: Antioxidant and Antifungal Properties of Cinnamon, Cloves, Melia azedarach L. and Ocimum gratissimum L. Extracts against Fusarium oxysporum Isolated from Infected Vegetables in Mauritius
Source: Pathogens. 2024 May 22;13(6):436. doi: 10.3390/pathogens13060436 (PMC11206713; doi:10.3390/pathogens13060436)
Supplement: Supplementary file 1 [file pathogens-13-00436-s001.zip › pathogens-2974466-supplementary.pdf]

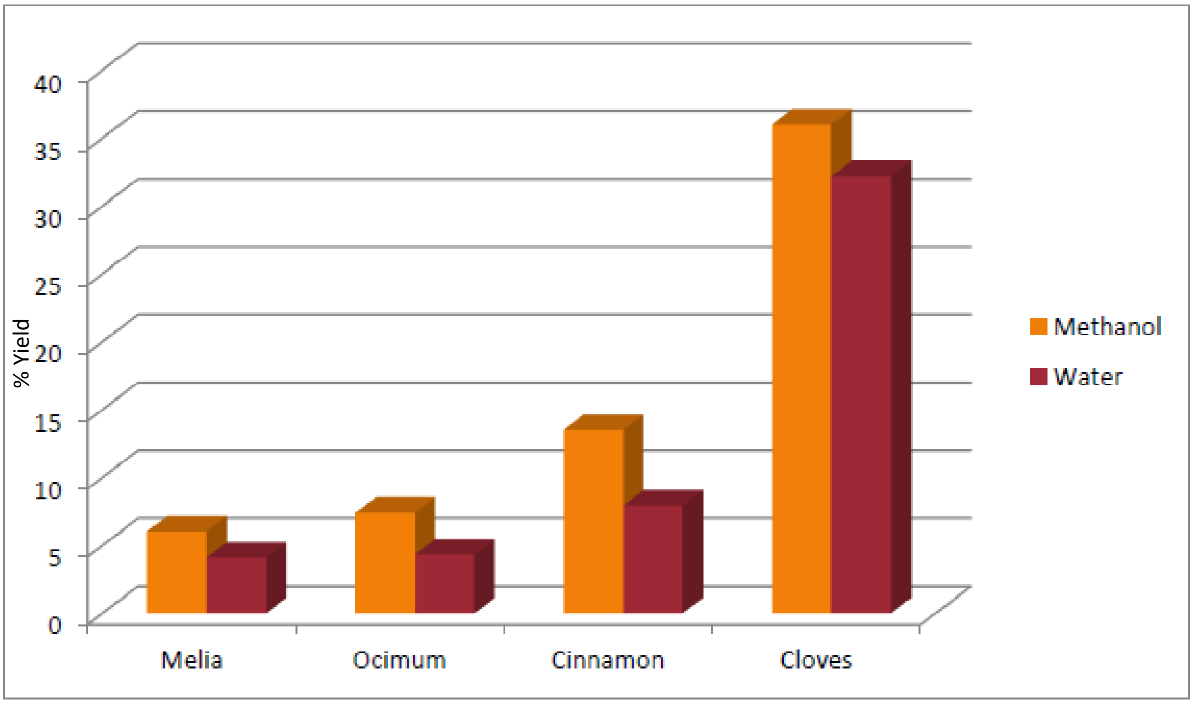

Figure S1: Extraction yield of different plants using methanol or water as solvent.

Table S1: Tests for the different constituents present in the extracts of cinnamon, cloves, *Meliaceae Melia azedarach L.* and *Ocimum gratissimum L.*

**Test**	**Method**	**Expected result**
Test for Alkaloids	To each extract, Mayer's reagent (potassium mercuric iodide) was added. Mayer's reagent was prepared by adding 0.36 g of mercuric chloride to 5.1 g of potassium iodide	The presence of alkaloids is confirmed when a yellow/cream precipitate was formed
Test for Flavonoids	Few drops of 20% sodium hydroxide were added with 2 mL of each extract, a dark yellow colour is observed. The yellow colour disappears by adding a few drops of 20% dilute hydrochloric acid to it.	Presence of flavonoids is confirmed by the formation and disappearance of the yellow color
Test for Saponins	6 mL of distilled water was added to 2 mL of each extract separately and shaken vigorously	Persistent foaming or formation of bubbles confirms the presence of saponins
Test for Tannins	1% of alcoholic ferric chloride was added to 2 mL of each extract	The presence of tannins is confirmed by the formation of black or brownish colour
Test for Phenols	2 mL of 5% aqueous ferric chloride was added to 2 mL of each extract	The presence of phenol is confirmed if a blue colour is formed in the sample extract
Test for Proteins	1 mL of 40% sodium hydroxide and few drops of 1% copper sulphate was added to 2 mL of each extract	The presence of peptide linkage molecules is confirmed if a violet colour appears in the sample extract
Test for Cardiac Glycosides	0.5 mL of glacial acetic acid and 3 drops of 1% aqueous ferric chloride solution were added to 1 mL of each extract	The presence of cardiac glycosides is confirmed if a brown ring is formed at the interface in the sample extract
Test for Terpenoids	To 1 mL of each extract add 0.5 mL of chloroform and few drops of concentrated sulphuric acid	The presence of terpenoids is confirmed if a reddish brown precipitate is formed in the extract
Test for Carbohydrates	Few drops of Molisch's reagent followed by 1 mL of concentrated sulphuric acid were added to the side of the tubes containing 2 mL of each extract.	After 2 to 3 minutes, the presence of carbohydrates is confirmed if a red or dull violet colour is formed in the sample extract

Table S2: Results of the survey to assess the prevalence of *Fusarium* infection in Mauritius fields.

Region	Affected crops	Visible symptoms	Season	Causative agent(s)
La Laura	Beans	Black rot	Summer	Fusarium
La Laura	Tomatoes, Potatoes	Fusarium wilts and black rot	Winter	*Fusarium oxysporum.*
La Laura	Tomatoes	Drying of leaves and black rot	Winter	*Fusarium oxysporum f.sp. lycopersici.*
La Laura	Tomatoes	Fusarium wilts	Summer	*Fusarium oxysporum.*
La Laura	Tomatoes	Black rot	Summer	*Fusarium oxysporum f.sp. lycopersici.*
La Laura	Tomatoes and Beans	Leaf scorch and Fusarium wilts	Winter	*Fusarium* and *Colletotrichum*
La Laura	Potatoes	Early and late blight disease	Winter	*Fusarium*
La Laura	Tomatoes	Leaf blight	Summer	*Fusarium oxysporum.*
Olivia	Tomatoes	Fusarium wilt and black rot	summer	*Fusarium oxysporum f.sp. lycopersici.*
Nouvelle Découverte	Tomatoes	Yellowing of leaves and wilting	Summer	*Fusarium oxysporum.*
Flacq	Tomatoes	Black rot	Summer	*Fusarium*
Palmar	Tomatoes	Yellowing of leafs at the base	Winter	*Fusarium oxysporum.*
Quartier Militaire	Tomatoes	Fusarium wilt and black rot	summer	*Fusarium oxysporum f.sp. lycopersici.*
Brahmstan	Tomatoes	Yellowing of leaves and wilting	Summer	*Fusarium oxysporum*
St Julien	Potatoes	Rotting and wilting of plants	Winter	*Fusarium*
Mont Ida	Tomatoes	Yellowing of leafs at the base	Summer	*Fusarium oxysporum.*
Camp Thorel	Tomatoes	Fusarium wilt and black rot	summer	*Fusarium oxysporum.*
L'esperance	Tomatoes	Yellowing of leaves and wilting	Summer	*Fusarium oxysporum f.sp. lycopersici.*
L'avenir	Beans	Leaf spots and black rot	Summer	*Fusarium* or *Colletotrichum*
St Pierre	Tomatoes	Leaf spots	Summer	*Fusarium oxysporum f.sp. lycopersici.*
Lesperance Trebuchet	Eggplant	Smaller fruits, reduced growth.	Winter	*Fusarium oxysporum* and fruitflies.
Lesperance Trebuchet	Tomato	Wiling and black rots on fruits.	Summer	*Fusarium oxysporum* and fruitflies.
Lesperance Trebuchet	Tomato	Yellowing on mature leaves and decay of fruits.	Summer	*Fusarium oxysporum* and fruitflies.
Mare Tabac	Tomato	Smaller fruits, reduced growth.	Summer	*Fusarium oxysporum*
Mare Tabac	Tomato	Yellowing of leaves at the base	Summer	*Fusarium oxysporum*
